# Consistent Quantification of Complex Dynamics via a Novel Statistical Complexity Measure

**DOI:** 10.3390/e24040505

**Published:** 2022-04-04

**Authors:** Frank Keul, Kay Hamacher

**Affiliations:** 1 Department of Biology, Technische Universität Darmstadt, 64289 Darmstadt, Germany; keul.frank@gmail.com; 2 Department of Physics, Technische Universität Darmstadt, 64289 Darmstadt, Germany; 3 Department of Computer Science, Technische Universität Darmstadt, 64289 Darmstadt, Germany

**Keywords:** complexity, co-evolution, Jensen–Shannon, entropy

## Abstract

Natural systems often show complex dynamics. The quantification of such complex dynamics is an important step in, e.g., characterization and classification of different systems or to investigate the effect of an external perturbation on the dynamics. Promising routes were followed in the past using concepts based on (Shannon’s) entropy. Here, we propose a new, conceptually sound measure that can be pragmatically computed, in contrast to pure theoretical concepts based on, e.g., Kolmogorov complexity. We illustrate the applicability using a toy example with a control parameter and go on to the molecular evolution of the HIV1 protease for which drug treatment can be regarded as an external perturbation that changes the complexity of its molecular evolutionary dynamics. In fact, our method identifies exactly those residues which are known to bind the drug molecules by their noticeable signal. We furthermore apply our method in a completely different domain, namely foreign exchange rates, and find convincing results as well.

## 1. Introduction

The dynamics of complex systems (complexity here refers to the notion used in physics and statistics, e.g., [1,2], not to other notions used, e.g., in design and manufacturing engineering [3]) is of great interest in all natural sciences. The most influential contribution of statistical physics to this field is the *quantification* beyond mere observation of the underlying mechanisms.

In particular, the effect of an external control parameter or an environmental factor on the internal dynamics reveals almost all technological exploitable insight for such a complex system. Examples range from the phenomenon of stochastic resonance and its applications [4,5,6] to ecological systems described by the well-known Lotka–Volterra-type models [7,8].

Thus, we are left with the question of how to consistently, transferably quantify the change in the dynamical complexity upon an external perturbation in a conceptually sound manner. To this end, we herein propose an information-theoretic measure and show its applicability with three distinct examples: (a) a toy problem based on the logistic equation as a complex, but for our purposes controllable, model; (b) the evolutionary response of the HIV1 protease to drug treatment; and (c) the dynamics of foreign exchange rates.

## 2. Methods

The overall behavior of an observable can be well characterized by the (discretized) state variable. The different states can enumerated by i∈[1,⋯,N], then the probability to observe the *i*-th value of the observable is $p_{i}$. The overall distribution of all $p_{i}$ can then be written in vector-form $\vec{p}$.

Wootters, Crutchfield, and Young [9,10] argued that a system which is (a) completely random ($p_{i}$ close to uniform) or (b) absolutely fixed (all but one $p_{i}$ vanish) should not be regarded as “complex”. Thus, complexity occurs between those edge cases.

For discrete probabilities stored in $\vec{p}=\left(p_{1},\cdots ,p_{i},\cdots ,p_{N}\right)$, both edge cases are reflected in Shannon’s entropy $H\left(\vec{p}\right):=-\sum_{i=1}^{N}p_{i}\cdot \log_{2}p_{i}$: for (a) we have a (close to) maximal entropy $\log_{2}N$ for data discretized into *N* bins or states, while for (b) we find a vanishing one. Both cases reflect non-complex behavior: either (a) non-correlated randomness or (b) absolute predictability. Thus, complex behavior is to be expected only at intermediate entropy values.

Often, *H* is normalized by its maximum $\log_{2}N$, so that $H\left(\vec{p}\right)=-\sum_{i}p_{i}\log_{N}p_{i}$, where *N* is the number of distinct states. (Note that the usage of Shannon’s entropy is related to the concept of “information content” in, e.g., [3], Equations (5.5) and (5.5a)). In a subsequent study [11], researchers developed the notation of Suh’s “information content” into an information-theory-based problem-solving methodology. The empirical counts $n_{i}$ are used to obtain maximum-likelihood estimators of the respective ${\hat{p}}_{i}=n_{i}/L$, where $L=\sum_{i}n_{i}$ is the number of all observations. Note that for “small” *L*, we would need to include corrections to the Shannon entropy formula to account for small coverage [12,13,14] for which efficient computational methods exist [15]. In this study, we deal with a synthetic dataset and two real-life ones that are sufficiently large so that we can avoid this complication here.

Earlier ideas on how to improve upon Shannon’s entropy to assess complexity (and thus work in the “intermediate” regime between vanishing and maximal entropy) by Wootters [9] were extended by several authors [16,17,18,19,20] into measures of the general form $M\left(\vec{p}\right):=H\left(\vec{p}\right)\cdot D\left(\vec{p},\vec{q}\right)$, where $D\left(\vec{p},\vec{q}\right)$ is a distance between the realized dynamics giving rise to $\vec{p}$ in relation to a reference distribution $\vec{q}$. The distribution $\vec{q}$ captures the dynamics of a system without the complex mechanism we are interested in. In all previous work [9,16,18,19,20], $\vec{q}$ was set to the uniform distribution $\forall i:u_{i}:=1/N$. Then, the measure *M* vanishes for completely random dynamics ($\vec{p}$ uniform, and thus $D\left(\vec{p},\vec{u}\right)\to 0$), as well as for constant values ($H\left(\vec{p}\right)\to 0$).

Below we will show that non-uniform distributions resulting from (reduced) complex dynamics are more informative to assess the response of a system to a perturbation or any change in its environment. Furthermore, the choice of *D* must not be ad hoc as in some of the previous work.

Note that an alternative approach to quantify the (stochastic) complexity of a system is based on using its Kolmogorov complexity [21]. While conceptually sound, this approach suffers from the fact that the Kolmogorov complexity is not analytically computable in general [22].

### 2.1. Complexity Change upon Perturbation

The well-known Kullback–Leibler divergence [23] compares two distributions in an information theoretic sense: $D_{KL}\left(\vec{p},\vec{q}\right):=\sum_{i}p_{i}\cdot \log\frac{p_{i}}{q_{i}}$ and was used in, e.g., [9] as the distance with respect to the uniform distribution $\vec{u}$.

The inherent asymmetry renders the $D_{KL}$ itself a non-metric. However, it can be symmetrized and becomes the Jensen–Shannon divergence

$$\begin{array}{ccc} D_{JS}\left(\vec{p},\vec{q}\right) & = & \frac{1}{2}\left(D_{KL}\left(\vec{p},\vec{m}\right)+D_{KL}\left(\vec{q},\vec{m}\right)\right)\\ \vec{m} & = & \frac{1}{2}\left(\vec{p}+\vec{q}\right) \end{array}$$

Now, $\sqrt{D_{JS}\left(\vec{p},\vec{q}\right)}$ was shown to fulfill all requirements of a metric [24].

Then, with regard to the previously used uniform distribution, we would obtain $M\left(\vec{p}\right):=H\left(\vec{p}\right)\cdot \sqrt{D_{JS}\left(\vec{p},\vec{u}\right)}$. In fact, this definition of stochastic complexity was already applied in quantitative finance [25,26] and general settings [27] within the Martín–Plastino–Rosso (MPR) framework for stochastic complexity.

Our extension revolves around the question of the *change* in complexity upon a perturbation rather than its absolute value itself. To compare two distinct scenarios—one before and one after an external perturbation—we compute the difference in those complexity values, where $\vec{p}$ is the distribution of an observable *X* before and $\vec{q}$ after a perturbation:

(1) $$\begin{array}{ccc} \Delta C\left(\vec{p}\right|\;\left|\vec{q}\right) & = & M\left(\vec{p}\right|\;\left|\vec{q}\right)-M\left(\vec{q}\right|\;\left|\vec{p}\right)\\ & = & H\left(\vec{p}\right)\sqrt{D_{JS}\left(\vec{p}\right|\;\left|\vec{q}\right)}-H\left(\vec{q}\right)\sqrt{D_{JS}\left(\vec{q}\right|\;\left|\vec{p}\right)}\\ & = & \left(H\left(\vec{p}\right)-H\left(\vec{q}\right)\right)\sqrt{D_{JS}\left(\vec{p}\right|\;\left|\vec{q}\right)} \end{array}$$

In the subsequent parts, we will use p(X) and $\vec{p}$ as well as p(Y) and $\vec{q}$ interchangeablely as *X* is our “reference” and *Y* our perturbed system. Note that we have replaced the uniform distribution put forward in previous work by the one of the unperturbed system. Therefore, we measure the *complexity change upon perturbation* by ΔC(X||Y).

### 2.2. Datasets

In order to assess the performance of ΔC(X||Y), we chose two distinct scenarios. First, we use a synthetic, controllable toy model which consists of coupled logistic maps in the chaotic regime with

(2) $$\begin{array}{ccc} x_{n+1} & = & f(x_{n})\\ y_{n+1} & = & \left(1-\xi \right)f\left(y_{n}\right)+\xi f\left(x_{n}\right)\\ f(x) & = & 4x(1-x) \end{array}$$

where 1≤n≤ 100,000 with the coupling ξ varying between 0 and 0.7. This system allows for the continuous modulation of the dynamics of $y_{n}$ by an external, independent dynamics, namely $x_{n}$. Note that *X* shows the same statistical properties as the unperturbed (ξ=0) *y*-system. We can thus use *X* in agreement with the nomenclature of the previous section.

Computation of ΔC (see below) was performed for the comparison of the distribution p(Y) with both the uniform *U* and the actually realized distribution of p(X). Note that in this case, the random variable serves two purposes at the same time: it is driving the perturbation to *Y* by the strength ξ, while the distributions $p\left(X\right)=p_{\xi =0}\left(Y\right)$ equal each other and thus p(X) can be regarded as the histogram of the unperturbed (ξ=0) dynamics of $\left\{y_{1},\cdots ,y_{n}\right\}$.

The second dataset consisted of protein sequences from the HIV protease (HIVP). This enzyme consists of 99 amino acids and is essential for the formation of functional HIV virions. It became one of the two major targets for drugs to treat AIDS. This dataset can be subdivided into sequences from patients treated and untreated by HIVP inhibitors. Then, we regard a treatment as a “perturbation” and we can gain insight into the reaction of the viral evolution to this evolutionary environmental change. Note that each of the 99 positions will be analyzed independently and regarded as a separated X↔Y pair.

The high number of annotated sequences [28] renders the HIVP an ideal area of application for ΔC(X||Y); furthermore, no multiple sequence alignment is necessary in this case—effectively avoiding poor signal-to-noise ratios (SNR), frequently encountered in biomolecular sequence studies [29]. To increase the SNR further, all sequences with more than one position with multiple and non-canonic amino acids were removed due to unknown combinatorics. The resulting 53,793 sequences were then subdivided into a *treated* (11,521 sequences) and *untreated* dataset (42,272 sequences). We computed ΔC between the distributions of *treated* and *untreated* sequences per position with the latter being the reference distribution. Note that for such a large dataset, we do not need to take into account finite-size corrections [30] as put forward by, e.g., Grassberger [13,14].

As a third example, we investigate the complex dynamics of foreign exchange markets (under external perturbation). A prime example is the change of those rates for the British Pound with respect to other currencies; here the external perturbation is the referendum (on 23 June 2016) to leave the European Union and thus (prospectively) reduce the strength of economic coupling of the British economy with other European countries. As the Pound was never part of the Euro system, the change in dynamics is *ceteris paribus* only affected by the decision alone. To this end, we obtained the tick data for the whole year 2016 from histdata.com for the currency pairs GBP-EUR, GBP-USD, GBP-CHF, and GBP-JYP. We averaged bit and ask prices to obtain one time series.

## 3. Results

We applied our measure ΔC(X||Y) to the datasets described above in the dataset section.

### 3.1. Synthetic Data Set for Coupled Logistic Maps

Using the synthetic dataset of the toy model in Equation (2), we illustrate the insight one might gain from ΔC(X||Y). Here, we discretized the continuous values simulated for the coupled map into 20 bins.

**Figure 1:**
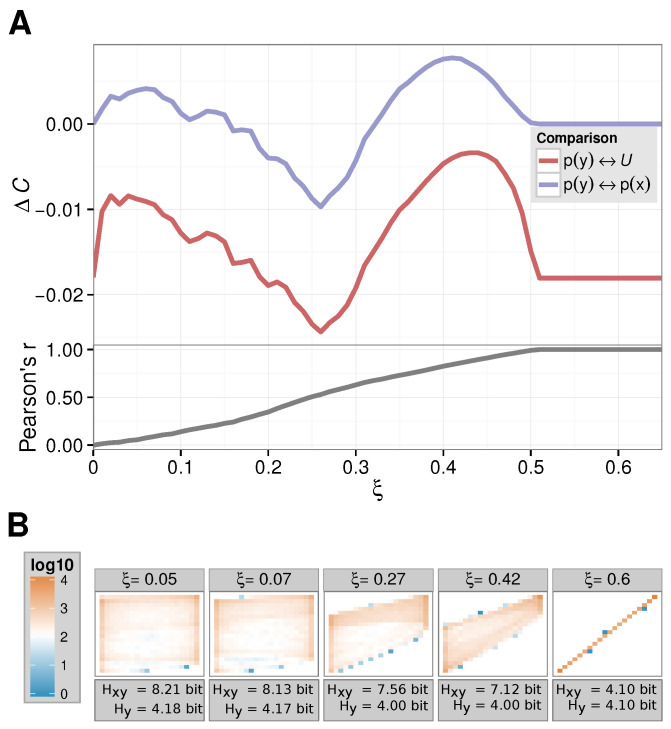
Coupled map: (**A**) complexity and correlation progression; (**B**) (Logarithmic) heatmap of the contingency tables/two-dimensional histograms p(X,Y) for varying ξ. For these ξ-values, ΔC(X||Y) reached its (local) maxima/minimum.

In Figure 1, we show our results. First, we show how the complexity of the dynamics changes upon varying ξ with respect to the uniform distribution (we set p(X)=U by hand, while recording the distribution p(Y) based on the simulated time series of Equation (2), red line in Figure 1A). We observe an undesirable effect: the ΔC vanishes neither for ξ=0, nor for large(r) ξ. However, in those cases, the complexity change induced by the perturbation should be regarded as small: for ξ=0 there is just no interaction, thus no change can occur. For large ξ, we can immediately conclude from Equation (2) that the dynamics of $y_{n}$ is actually equivalent to the one of $x_{n}$, so it is again the dynamics of one logistic equation. This is due to the fact that perfect synchronization between the two processes occurs only at intermediate coupling strengths, as Sun et al. [31] already discussed. This argument is further strengthened by the Pearson correlation coefficient between $x_{n}$ and $y_{n}$ that we also show in Figure 1A: for ξ≥0.5 the time series correlated perfectly; namely, they are the same. This can also be seen in the two-dimensional histogram of $\left(x_{n},y_{n}\right)$ value pairs for ξ=0.6 recorded for Figure 1B.

In Figure 1A, we also show the ΔC values computed as above (blue line). First we note that the sensible results for ξ=0 and ξ>0.5 eventually are produced. ΔC(X||Y) actually vanishes at precisely ξ=0.5, at which $x_{n}$ and $y_{n}$ are synchronized.

Furthermore, we observe that Shannon’s entropy of the observable $H_{Y}$ alone does not allow for any insight into the complexity (change) of the dynamics of $y_{n}$ as it remains within an error margin for varying ξ. Furthermore, note that Shannon’s entropy $H_{XY}$ for the two-dimensional histogram p(X,Y) continuously decreases for larger couplings ξ as the distributions become more and more spiked.

In addition, our ΔC(X||Y) shows a “direction” in the sense that an increase in complexity (or a reduction) can be detected. For ξ=0.27, one can, e.g., clearly observe a stronger connection between *X* and *Y* in the p(X,Y) of Figure 1A; thus, a reduction in the complexity – $H\left(X\right)=H\left(\vec{q}\right)$ dominates the first factor in Equation (1). At ξ≈0.07 and ξ=0.42, we found the exact opposite: here, the entropy of H(Y) reaches a comparable low value and thus influences the ΔC accordingly.

Note that for other information theoretic measures such as the mutual information MI(X,Y):=H(X)+H(Y)−H(X,Y), we obtain less sensible results. For this measure, we have H(X)≈4.14 bit=const for all ξ. We then would obtain a monotonously increasing MI running in analogy to Pearson’s correlation coefficient.

### 3.2. Complexity within HIVP

We proceeded to apply ΔC(X||Y) to each position in the HIVP sequences described above. For each position, we can therefore quantitatively assess the evolutionary impact of drug treatment. Here, we built histograms as counts for each of the 20 naturally occurring amino acid types. *X* then are the (amino acid) outcomes for the untreated patient dataset, while *Y* are the ones for the treated patients for which the viral evolution is under-perturbed selective pressure.

**Figure 2:**
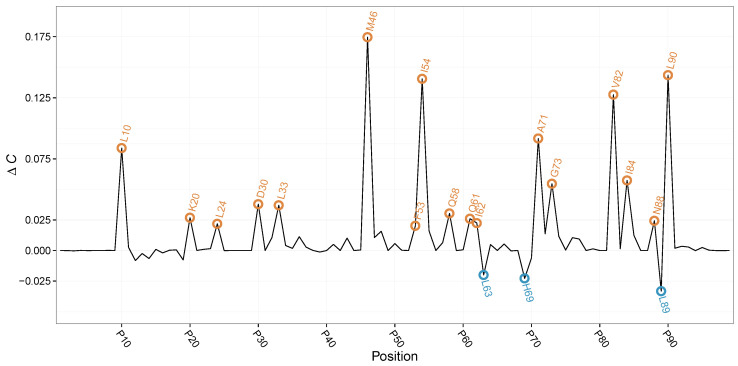
ΔC values for the HIVP positions. In orange, peaks with $\Delta C\geq 0.1\cdot \max\left(\Delta C\right)$ are highlighted, whereas peaks with $\Delta C\leq -0.1\cdot \max\left(\Delta C\right)$ are shown in blue. Almost all peak positions have been reported to be influenced by protease inhibitors.

By ΔC, we were able to identify previously reported compensatory mutation by setting a threshold at $\left|\Delta C\right|\geq 0.1\cdot \max\left(\Delta C\right)$. Here, two different groups of ΔC can be observed as ΔC can be either positive or negative in value (see Figure 2). Hence, ΔC allows us not only to annotate position in HIVP where the amino acid distributions between *treated* and *untreated* differ, but also allows us to annotate the direction in change of evolutionary complexity.

For positive ΔC, we find positions 10, 20, 24, 30, 33, 46, 53, 54, 58, 62, 71, 73, 82, 84, 88, and 89. Earlier studies revealed a reduced susceptibility to protease inhibitors at these positions [32,33] (see Table 1). Only position 61 has yet to be reported as compensatory mutation upon protease inhibitor administration. For the aforementioned position, we observe a positive ΔC, resulting in increased evolutionary complexity upon protease inhibitor treatment. Here, HIVP increases the amino acid diversity to compensate the drug-induced evolutionary pressure. Interestingly, we observe increased frequencies of smaller, polar amino acids at position 61 upon drug treatment, indicating a potentially not documented compensatory effect.

For the second group of ΔC, positions with a negative ΔC, we obtain positions 63, 69, and 89, with all three reported earlier to be affected by drug treatment [33]. The negative ΔC at these sites points to a reduction in position-wise evolutionary complexity upon treatment with protease inhibitors, indicating ideal targets for further combination therapy.

### 3.3. Foreign Exchange Rates under Perturbation

As described above, we used the foreign exchange rate of the pound sterling to other major currencies and obtained the differences of our complexity measure before and after the Brexit referendum. As mentioned above, we used the average of bid and ask prices. We downloaded the tick data for the year 2016 from histdata.com and thus have market data roughly from half a year before and half a year after the referendum (the Brexit referendum was held on 23 June 2016). We binned the rates, following standard procedure, into basis points (bps).

**Figure 3:**
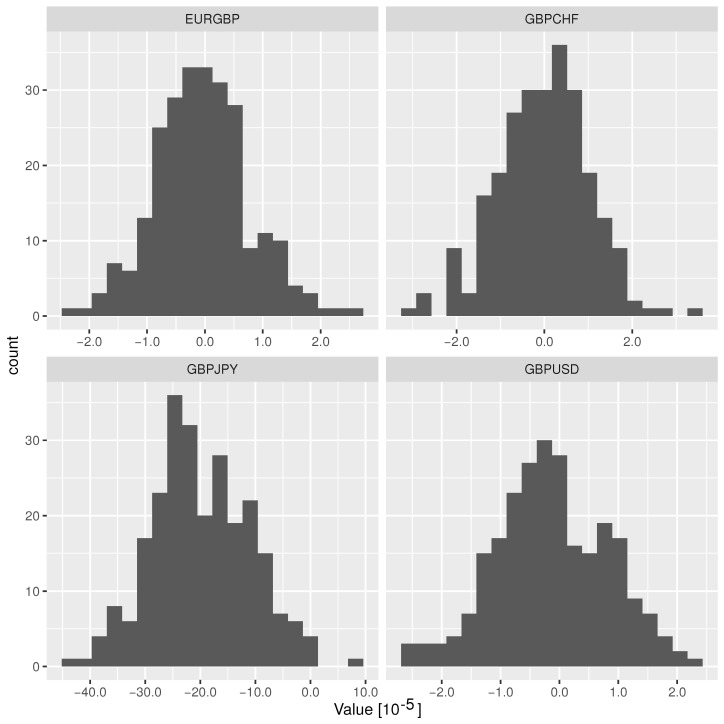
Histograms of ΔC for the currency pairs under investigation obtained for resampled data.

In Table 2, we summarize the findings. To assess the statistical relevance of the ΔC values in Table 2, we additionally applied a resampling technique. We sampled 250 replicas of the original data into random temporal order, extracting the same number of data points as in the original dataset for the sets of exchange rates before and after the referendum. Then, we computed the ΔC values for these as an in-sample estimator of the ΔC values regardless of intervention or external perturbation. The resulting histograms are shown in Figure 3. This allows us to judge the original values with respect to an ensemble that contains no signature of the referendum in the spirit of similar approaches to resampling [34,35,36]. To this end, for each currency pair we computed the *Z*-value under resampling, where $Z:=\frac{\Delta C-\mu }{\sigma }$ with μ and σ being the mean and the standard deviation over the 250 samples for each currency pair.

Under a two-sided test—the question being whether the ΔC values for the raw data are outliers to the resampled ones—all currency pairs show a significant signal. This can already be seen from the standard deviation of the resampled data in Figure 3, which turns out to be of the order $10^{-6}$ to $10^{-7}$.

Our results suggest a complex scenario that economically makes sense. In particular, the foreseeable to-be-expected de-coupling of the United Kingdom’s economy from the one of the EU strengthens the independence towards the Euro (positive entropy difference in Equation (1)) which can be seen in the positive ΔC of Table 2. The US Dollar and the Euro (as the two major currencies) are tightly coupled, so that the positive ΔC for the Pound–Dollar exchange rate might be an indirect effect due to overall Euro influence. Furthermore, the negative ΔC values for GBP-CHF and GBP-JPY are the flip side of the same coin: while the complexity of the GBP-EUR and GBP-USD dynamics was somewhat related and became less tightly coupled after the referendum, it is a logical necessity that the GBP-*xyz* (where *xyz* stand for any other economic entity) dynamics get closer (negative ΔC) to each other.

## 4. Discussion

In this study, we have derived an information theoretic measure ΔC(X||Y) to quantitatively assess the impact of an external perturbation on the complexity of a dynamical system. Starting from previous work, such as the MPR framework [19,27], we derived this measure based on a set of requirements.

For a controllable toy system, we showed that ΔC(X||Y) fulfills the requirements and delivers reasonable results.

In a real application, we assessed the viral evolution of the HIV1 protease under the influence of drug treatment targeting this particular enzyme. Our measures identified—purely based on the biomolecular sequences—those positions that were identified in expansive experiments as the ones to which the particular drugs bind. Interestingly, we can also extract signals on the type of drug used in any treatment.

Furthermore, our measure was able to show the impact on economic time series (foreign exchange rates) upon stress (Brexit referendum).

We note in passing that the *qualitative* results of our analysis were the same for both $\sqrt{D_{JS}\left(\vec{p},\vec{q}\right)}$ and $D_{JS}\left(\vec{p},\vec{q}\right)$, non-surprisingly due to the monotonicity of the square root.

## Figures and Tables

**Table 1:** Summary of positions mutated by the four most prominent protease inhibitors in the HIVP dataset. **Bold** positions show a $\left|\Delta C\right|\geq 0.1\cdot \max\Delta C$. Underlined positions represent “major” HIVP mutations which are detected to mutate first in presence of a drug.

Drug	Number of Sequences	Affected Positions
Indinavir	3753	**10**, **20**, **24**, 32, 36, **46**, **54**, **71**, **73**, 76, **82**, **84**, **89**
Lopinavir	955	**10**, **20**, **24**, 32, **33**, **46**, 47, 50, **53**, **54**, **63**, **71**, **73**, 76, **82**, **84**, **90**
Nelfinavir	3178	**10**, **30**, 36, **46**, **71**, 77, **82**, **84**, **88**, **90**
Saquinavir	2526	**10**, **24**, 48, **54**, **62**, **71**, **73**, 77, **82**, **84**, **90**

**Table 2:** Our complexity measure for various exchange rate distributions. Here, $p_{a}$, $p_{b}$, and $p_{u}$ are the distributions of the exchange rates before and after the Brexit referendum and the uniform distribution, respectively. Clearly, ΔC is always negative with respect to the uniform distribution $p_{u}$, as the entropy of $p_{u}$ is maximal; thus, ΔC can only decrease. Note, however, the amount of decrease differs widely. To assess the significance, we performed a permutation test and calculated the *Z*-score for $\Delta C\left(p_{a}\left|\right|p_{b}\right)$ (see main text for details).

Currency Pair	$\Delta C\left(p_{a}\left|\right|p_{b}\right)$	$\Delta C\left(p_{b}\left|\right|p_{u}\right)$	$\Delta C\left(p_{a}\left|\right|p_{u}\right)$	*Z*
GBP-EUR	0.207	−0.455	−0.60	$252\cdot 10^{3}$
GBP-USD	0.449	−0.387	−0.821	$468\cdot 10^{3}$
GBP-CHF	−0.0247	−0.493	−0.482	$-23\cdot 10^{3}$
GBP-JPY	−0.115	−0.503	−0.398	$-12\cdot 10^{3}$

## Data Availability

Not applicable.

## References

[1] Wolfram S. (1994). Cellular Automata and Complexity—Collected Papers.

[2] Alligood K.T., Sauer T.D., Yorke J.A. (1997). Chaos: An Introduction to Dynamical Systems.

[3] Suh N.P. (1990). The Principles of Design.

[4] Gammaitoni L., Hänggi P., Jung P., Marchesoni F. (1998). Stochastic resonance. Rev. Mod. Phys..

[5] Shi N., Ugaz V.M. (2014). Entropic stochastic resonance enables trapping under periodic confinement: A Brownian-dynamics study. Phys. Rev. E.

[6] McDonnell M.D., Stocks N.G., Pearce C.E.M., Abbott D. (2008). Stochastic Resonance–From Suprathreshold Stochastic Resonance to Stochastic Signal Quantization.

[7] Lotka A.J. (1910). Contribution to the theory of periodic reactions. J. Phys. Chem..

[8] Volterra V., Ferrari C. (1927). Variazioni e Fluttuazioni del Numero D’individui in Specie Animali Conviventi.

[9] Wootters W.K. (1981). Statistical distance and Hilbert space. Phys. Rev. D.

[10] Crutchfield J.P., Young K. (1989). Inferring statistical complexity. Phys. Rev. Lett..

[11] Fan L., Cai M., Lin Y., Zhang W. (2015). Axiomatic design theory: Further notes and its guideline to applications. Int. J. Mat. Prod. Techn..

[12] Grassberger P. (2008). Entropy Estimates from Insufficient Samplings. arXiv.

[13] Grassberger P. (1988). Finite sample corrections to entropy and dimension estimates. Phys. Lett. A.

[14] Holste D., Grosse I., Herzel H. (1998). Bayes’ estimators of generalized entropies. J. Phys. A Math. Gen..

[15] Stammler S., Katzenbeisser S., Hamacher K., Domingo-Ferrer J., Pejić-Bach M. (2016). Correcting Finite Sampling Issues in Entropy l-diversity. Proceedings of the Privacy in Statistical Databases: UNESCO Chair in Data Privacy, International Conference, PSD 2016.

[16] Martin M., Plastino A., Rosso O. (2003). Statistical complexity and disequilibrium. Phys. Lett. A.

[17] Ricardo Lopez-Ruiz H.M., Calbet X., Kowalski A.M., Rossignoli R.D., Curado E.M.F. (2013). A Statistical Measure of Complexity. Concepts and Recent Advances in Generalized Information Measures and Statistics.

[18] Feldman D.P., Crutchfield J.P. (1998). Measures of statistical complexity: Why?. Phys. Lett. A.

[19] Martin M., Plastino A., Rosso O. (2006). Generalized statistical complexity measures: Geometrical and analytical properties. Phys. A Stat. Mech. Its Appl..

[20] Kowalski A.M., Martín M.T., Plastino A., Rosso O.A., Casas M. (2011). Distances in Probability Space and the Statistical Complexity Setup. Entropy.

[21] Emmert-Streib F. (2010). Statistic Complexity: Combining Kolmogorov Complexity with an Ensemble Approach. PLoS ONE.

[22] Vitanyi P.M. (2020). How Incomputable Is Kolmogorov Complexity?. Entropy.

[23] Cover T.M., Thomas J.A. (2006). Elements of Information Theory.

[24] Endres D.M., Schindelin J.E. (2003). A new metric for probability distributions. IEEE Trans. Inf. Theory.

[25] Ribeiro H.V., Jauregui M., Zunino L., Lenzi E.K. (2017). Characterizing time series via complexity-entropy curves. Phys. Rev. E.

[26] Bariviera A.F., Zunino L., Rosso O.A. (2017). Crude oil market and geopolitical events: An analysis based on information-theory-based quantifiers. Fuzzy Econ. Rev..

[27] Rosso O., Zunino L., Pérez D., Figliola A., Larrondo H., Garavaglia M., Martín M., Plastino A. (2007). Extracting features of Gaussian self-similar stochastic processes via the Bandt-Pompe approach. Phys. Rev. E.

[28] Rhee S.Y., Gonzales M.J., Kantor R., Betts B.J., Ravela J., Shafer R.W. (2003). Human immunodeficiency virus reverse transcriptase and protease sequence database. Nucleic Acids Res.

[29] Hess M., Keul F., Goesele M., Hamacher K. (2016). Addressing inaccuracies in BLOSUM computation improves homology search performance. BMC Bioinform..

[30] Weil P., Hoffgaard F., Hamacher K. (2009). Estimating Sufficient Statistics in Co-Evolutionary Analysis by Mutual Information. Comput. Biol. Chem..

[31] Sun J., Bollt E.M. (2014). Causation entropy identifies indirect influences, dominance of neighbors and anticipatory couplings. Phys. D Nonlinear Phenom..

[32] Rhee S.Y., Taylor J., Fessel W.J., Kaufman D., Towner W., Troia P., Ruane P., Hellinger J., Shirvani V., Zolopa A. (2010). HIV-1 Protease Mutations and Protease Inhibitor Cross-Resistance. Antimicrob. Agents Chemother..

[33] Wensing A.M., Calvez V., Günthard H.F., Johnson V.A., Paredes R., Pillay D., Shafer R.W., Richman D.D. (2014). 2014 Update of the drug resistance mutations in HIV-1. Top Antivir. Med..

[34] Boba P., Bollmann D., Schoepe D., Wester N., Wiesel J., Hamacher K. (2015). Efficient computation and statistical assessment of transfer entropy. Front. Phys..

[35] Waechter M., Jaeger K., Thuerck D., Weissgraeber S., Widmer S., Goesele M., Hamacher K. (2014). Using graphics processing units to investigate molecular coevolution. Concurr. Comput. Pract. Exp..

[36] Hamacher K. (2008). Relating Sequence Evolution of HIV1-Protease to Its Underlying Molecular Mechanics. Gene.

